# A Treatise on the Practice of Medicine

**Published:** 1852-08

**Authors:** 


*A Treatise on the Practice of Medicine.* By GEORGE B. WOOD, M. D., Prof. of the Theory and Practice of Medicine in the University of Pennsylvania, etc., etc., etc. Third Edition. In two volumes. Philadelphia: Lippincott, Grambo & Co., No. 14 North Fourth Street. 1852.

The success of this treatise, evidenced by the call for a third edition, shows, in the first place, the acceptableness of works of that description to medical students and practitioners. Their convenience and value, indeed, cannot be doubted; and yet, they are not without their disadvantages. Presenting a compendium of the contributions to science which are scattered among diverse publications, the reader is apt to be satisfied without going to the original fountains whence the authors derive a large share of their materials. So far as treatises on practice are compilations (which they usually are for the most part) the result just mentioned is an evil offsetting, in some measure, their good effects. Another evil is, that readers are apt to look to a popular practical work too much in the light of an authority, and to attach the same importance to the notions which the author may enunciate on his own responsibility, as to the opinions or doctrines which he appropriates from others. It does not require an argument to prove that a person may be well fitted to systematize and condense the knowledge which has been rendered available by the numerous laborers in the field of science, while his personal authority is entitled to but little weight. For the one, industry and tact are alone requisite; the other involves opportunities and faculties which do not follow as a matter of course.

But, in the second place, the success of Dr. Wood's work shows that, in the estimation of the medical public, it is well adapted to fulfill the objects for which it was prepared. We have heretofore expressed our opinion of its character. It bears internal evidence of care, and industry, and contains the most approved modern views and doctrines relating to the various subjects embraced in practical medicine. As such it commends itself to the continued approbation of the profession. That the principles and rules of practice which the learned author inculcates are in all instances unexceptionable, we are by no means willing to admit. We are free to say that we find in the two volumes much that, as a practitioner, we cannot adopt; but after saying this, we are not inconsistent when we avow, in our critical capacity, the opinion that it is well adapted to serve as a text book and work of reference, in other words a repository of current facts and opinions, which deserves to be studied by the medical novitiate, and consulted by the practicing physician, but not to supersede special treatises, nor to be taken as a guide, to the prejudice of independent thought and investigation.

The present edition has been enlarged by the addition of nearly eighty pages. The author remarks in the preface that "the reader will find evidences of modifications of the treatise scattered here and there throughout the work. Among the additions may be mentioned brief notices of several diseases, which, from their rarity, or total absence in this country, or from being unknown as distinct affections, were not described in the former editions. Such are the relapsing fever of Dr. Jenner, the leucocythemia of Professor Bennet, the dengue, and certain cutaneous affections, as trichosis, lupus, and pellagra. The chief modifications, however, have reference to subjects previously more or less discussed, as, for example, to inflammation, fatty degeneration, careinoma, epidemic cholera, the treatment of phthisis, the nature of hemorrhage, Bright's disease, etc."

